# Profiling and Quantification of Anthocyanins in Purple-Pericarp Sweetcorn and Purple-Pericarp Maize

**DOI:** 10.3390/molecules28062665

**Published:** 2023-03-15

**Authors:** Apurba Anirban, Hung T. Hong, Tim J. O’Hare

**Affiliations:** 1 Centre for Nutrition and Food Sciences, Queensland Alliance for Agriculture & Food Innovation (QAAFI), The University of Queensland, Brisbane, QLD 4072, Australia; 2 Collaborative Research Space, School of Agriculture and Food Sciences, University of Queensland, Gatton, QLD 4343, Australia

**Keywords:** anthocyanin, cyanidin, pelargonidin, malonylation, starchy maize, purple sweetcorn, white sweetcorn, *shrunken2*

## Abstract

Purple-pericarp sweetcorn accessions, derived from crossing purple-pericarp maize with white *shrunken2* sweetcorn, were assessed for differences in anthocyanin profile at both sweetcorn eating stage and at full kernel maturity. The ‘Tim1’ sweetcorn line developed a similar total anthocyanin concentration to its ‘Costa Rica’ parent when assessed at sweetcorn-eating stage. At full maturity it surpassed the purple maize parent, but this was mainly due to the presence of starch diluting the anthocyanin concentration of the latter. The anthocyanin/colour relationship was affected by both total anthocyanin concentration and the ratio of cyanidin- to pelargonidin-based anthocyanins. Malonylation of anthocyanins was also found to vary and did not appear to be linked with either cyanidin:pelargonidin ratio or total anthocyanin concentration. In addition, anthocyanin synthesis was affected by kernel maturity at harvest, with colour development increasing in conjunction with a progression of anthocyanin development across the kernel surface. Pigmentation was present in the aleurone, pericarp and vitreous endosperm of kernels of the purple-pericarp maize parent and purple-pericarp sweetcorn accessions when fully mature, but pigmentation was only apparent in the pericarp at sweetcorn-eating stage. Importantly for consumers, anthocyanin pigmentation covered almost the entire kernel surface at sweetcorn-eating stage.

## 1. Introduction

Purple sweetcorn based on the recessive supersweet mutation, *shrunken2* (*sh2*), is uncommon due to an extremely tight genetic linkage between *sh2* and a non-functional allele (*a1*) of the anthocyanin biosynthesis gene, *anthocyaninless1* [1,2]. Conversely, in starchy purple maize (*A1A1.Sh2Sh2*), the functional allele for starch biosynthesis (*Sh2*) is linked to a functional allele (*A1*) of *anthocyaninless1* [3]. As a result, the kernels of starchy purple maize are purple, round (unshrunken) and not sweet, due to the presence of starch, rather than sugar, while the kernels of sweetcorn are non-purple and shrunken, due to the absence of anthocyanin and starch, respectively, when fully mature. Recently, a novel purple *sh2* sweetcorn was developed from a Peruvian purple maize (‘Costa Rica’) and a white *sh2* sweetcorn (‘Tims-white’) by breaking the tight genetic link between *a1* and *sh2* [4,5].

The appearance of the purple colour in maize kernels is due to the synthesis of anthocyanin in the pericarp tissue and/or in the aleurone [6]. Sweetcorn lacks any anthocyanin pigmentation in these outer layers of the kernel, resulting in a translucent pericarp, such that the colour of the underlying endosperm gives sweetcorn its characteristic yellow or white colour. In pericarp-pigmented maize, the anthocyanin concentration is eight-fold higher than that found in aleurone-pigmented maize [7], making pericarp-coloured sweetcorn a better dietary source of anthocyanin. As with aleurone-pigmented maize, anthocyanin in the purple-pericarp sweetcorn kernel increases in concentration with increasing kernel maturity [8]. However, in the latter, anthocyanin development begins at the point of silk attachment, gradually covering the surface of the kernel as the kernel matures [9]. Anthocyanins have been previously identified in starchy purple maize and purple *brittle1* sweetcorn as being comprised of anthocyanin derivatives of cyanidin, peonidin and pelargonidin [10], which are often malonylated [8,11].

Apart from providing a novel colour to sweetcorn, anthocyanin has also been associated with a number of nutritional attributes, both in animal and human studies. Different reviews [12,13] have indicated that purple maize anthocyanins have nutritional and health benefits. Peruvian-purple maize anthocyanin extracts have been reported to reduce hypertension in humans [14] and inhibit colorectal carcinogenesis in male rats [15]. Anthocyanin is also used as a natural food colourant [16], although its stability during processing can vary, and can be impacted by the degree of acylation of the anthocyanin molecule [17]. Purple maize is reported to be a rich source of anthocyanins, particularly cyanidin and pelargonidin, and offers a natural alternative to synthetic dyes within the red to reddish-purple colour range [18].

Sweetcorn is harvested and eaten when the kernel is still physiologically immature [19], in contrast to purple-pericarp maize that is used for flour or food colouring [20]. Consequently, the anthocyanin concentration and profiles in purple-pericarp sweetcorn is likely to be potentially different from purple-pericarp maize. As the former is a novel product, the anthocyanin profile has not been previously analysed, either at the normal sweetcorn eating stage or at its full-kernel maturity. It is expected that the anthocyanin profile of the purple sweetcorn would be similar at both maturity stages to its ‘Costa Rica’ purple maize parent. As such, the primary aim of the current study was to document total anthocyanin concentration and the variation in proportion of individual anthocyanins of the different lines, and how these compare to the parent lines, at both sweetcorn eating stage and at full kernel maturity.

## 2. Results

### 2.1. Anthocyanin Quantification of Mature Round (Starchy) Kernels

Total anthocyanin concentration of mature kernels of purple-pericarp, ‘Costa Rica’ was significantly higher (194.47 mg/100g FW) than the purple-aleurone maize (‘Tims-aleurone’) (27.65 mg/100g FW) (Table 1). There was no anthocyanin detected in the white shrunken ‘Tims-white’ parental line. Cyanidin-, peonidin- and pelargonidin-based anthocyanin compounds were identified by UHPLC-DAD-MS at 60 DAP (day after pollination). Significant difference (*p* < 0.05) existed between ‘Costa Rica’ and ‘Tims-aleurone’ regarding cyanidin and peonidin concentration; however, pelargonidin concentration was not significantly different (Table 1). The anthocyanin profile of ‘Costa Rica’ consisted of 97% cyanidin (with peonidin) and 3% pelargonidin. By contrast, the ‘Tims-aleurone’ genotype had 90% cyanidin (with peonidin) and 10% pelargonidin.

### 2.2. Anthocyanin Quantification of Mature Shrunken (Non-Starchy) Kernels

The anthocyanin concentrations of the F3 mature shrunken sweetcorn segregants (‘Tim1’, ‘Tim2’, ‘Tim4’ and ‘Tim5’) at 60 DAP were found to differ significantly (Table 1). The highest total anthocyanin concentration (1109.3 mg/100g FW) was observed in the ‘Tim2’ purple shrunken genotype, which was significantly higher (*p* < 0.05) than the other ‘Tim’ segregants (Table 1). ‘Tim1’, ‘Tim4’ and ‘Tim5’ segregants contained 365.4, 335.3 and 649.3 mg/100g FW total anthocyanin, respectively, in which the ‘Tim1’ and ‘Tim4’ were not significantly different (*p* < 0.05) from each other. Similarly, there were significant differences (*p* < 0.05) in cyanidin, peonidin and pelargonidin concentrations between the individual ‘Tim’ lines, but there was no significant difference between ‘Tim1’ and ‘Tim4’, except for peonidin (Table 1). The ‘Tim2’ and ‘Tim5’ anthocyanin profiles consisted of 91% cyanidin (with peonidin) and 9% pelargonidin, whereas the ‘Tim1’ and ‘Tim4’ genotypes consisted of 82% cyanidin (with peonidin) and 18% pelargonidin, respectively.

### 2.3. Quantification of Anthocyanin at Eating-Stage (25 DAP) of Parental and F6 ‘Tim’ Purple Sweetcorn Accessions

**Figure 1:**
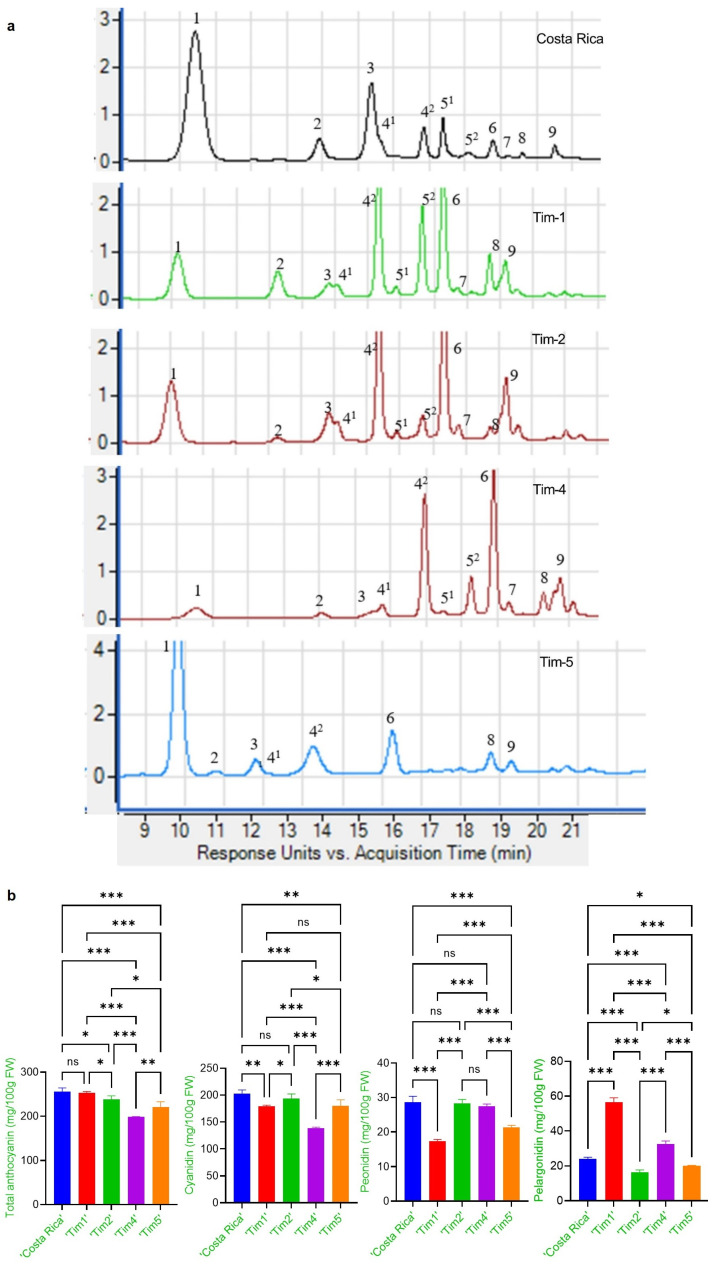
(**a**) Anthocyanin chromatograms of the ‘Costa Rica’ parent and developed ‘Tim’ F6 sweetcorn lines at 25 DAP obtained from UHPLC-DAD-MS at 520nm, with peaks (1) cyanidin-3-glucoside, (2) pelargonidin-3-glucoside, (3) peonidin-3-glucoside, (4^1^) cyanidin-3-malonyl glucoside, (4^2^) cyanidin-3-malonyl glucoside isomer, (5^1^) pelargonid-in-3-malonyl glucoside, (5^2^) pelargonidin-3-malonyl glucoside isomer, (6) cyanidin-3-dimalonyl glucoside, (7) peonidin-3-malonyl glucoside, (8) pelargonidin-3-dimalonyl glucoside, (9) peonidin-3-dimalonyl glucoside; (**b**) Fresh weight concentration of total anthocyanin and cyanidin-, peonidin- and pelargonidin-based anthocyanins of ‘Costa Rica’ and the ‘Tim’ F6 sweetcorn lines at 25 DAP. ns = non-significant, * = significant at *p* < 0.05, ** = significant at *p* < 0.01, and *** = significant at *p* < 0.001).

Analysis of anthocyanin derivatives at the sweetcorn eating stage (25 DAP) identified nine cyanidin-, peonidin- and pelargonidin-based (Figure 1a) anthocyanins in the F6 ‘Tim’ purple-pericarp sweetcorn accessions, differing in their degree of malonylation. These included cyanidin-3-glucoside (Cy3G), cyanidin-3-malonyl glucoside (Cy3MG), cyanidin-3-dimalonyl glucoside (Cy3DMG); peonidin-3-glucoside (Pn3G), peonidin-3-malonyl glucoside (Pn3MG), peonidin-3-dimalonyl glucoside (Pn3DMG), pelargonidin-3-glucoside (Pg3G), pelargonidin-3-malonyl glucoside (Pg3MG), and pelargonidin-3-dimalonyl glucoside (Pg3DMG) (Table 2). The identification of anthocyanins in purple sweetcorn was identified according to Hong et al. 2021 [9].

It was found that the sweetcorn purple-pericarp line ‘Tim1’ (F6) produced 253.0 mg/100g FW total anthocyanin at 25 DAP, in comparison to its purple maize parental line ‘Costa Rica’ (255.8 mg/100g FW), which was not significantly different (*p* < 0.05) (Figure 1b). Again, there was no anthocyanin detected in the white sweetcorn parent, ‘Tims-white’ at 25 DAP. Total anthocyanin concentration observed in ‘Tim2’, ‘Tim4’ and ‘Tim5’ was 238.1, 198.7, and 221.4 mg/100g FW, respectively, which were all significantly lower than ‘Tim1’ (*p* < 0.05) (Figure 1b).

The main anthocyanins identified in all purple lines were cyanidin-based glucosides. Although there was no significant difference between ‘Tim1’ and ‘Costa Rica’ regarding total anthocyanin concentration at 25 DAP, significant differences in cyanidin-, peonidin- and pelargonidin-based glucoside concentrations existed (Figure 1b). There was no significant difference (*p* < 0.05) between ‘Costa Rica’ and ‘Tim2’, or between ‘Tim1’ and ‘Tim5’ regarding cyanidin concentration, whereas a significant difference of pelargonidin-based concentration was observed in all lines (Figure 1b). At sweetcorn eating-stage, the anthocyanin profiles of eating-stage kernels of ‘Çosta Rica’, ‘Tim1’, ‘Tim2’, ‘Tim4’ and ‘Tim5’ consisted of 91%, 78%, 93%, 84% and 91% cyanidin-based (with peonidin-based) and 9%, 22%, 7%, 16% and 9% pelargonidin-based, respectively.

The proportion of anthocyanin malonylation was also observed to vary between the ‘Costa Rica’ purple maize parent and the individual ‘Tim’ lines at 25 DAP (Table 2, Figure 1a). The lowest proportion of malonylation was observed in the ‘Costa Rica’ parent and ‘Tim5’, where only 36.0% and 29.5% of anthocyanins, respectively, were malonylated. The highest proportion of malonylation (87.5%) occurred in ‘Tim4’, while ‘Tim1’ and ‘Tim2’ had intermediate levels (73.3% and 72.3%, respectively). The degree of malonylation appeared to be independent of the cyanidin- to pelargonidin-based ratio, with the higher pelargonidin line ‘Tim1’ and the lowest line ‘Tim2’ exhibiting a similar proportion of malonylation.

### 2.4. Objective Colour Measurement of the Mature Round (Starchy) and Shrunken (Non-Starchy) Kernels

**Figure 2:**
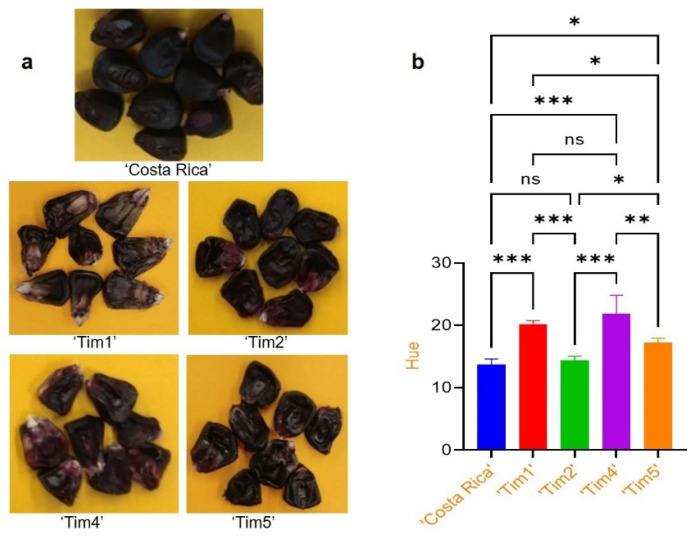
Objective colour measurement of mature kernels: (**a**) mature round kernels of ‘Costa Rica’ and F3 purple shrunken kernels (‘Tim1’, ‘Tim2’, ‘Tim4’ and ‘Tim5’); (**b**) comparative analysis of hue values of mature kernels of ‘Costa Rica’ and mature F3 shrunken kernels. Multiple comparison indicating ns = non-significant, * = significant at *p* < 0.05, ** = significant at *p* < 0.01 and *** = significant at *p* < 0.001.

The hue (H*) values of the mature round kernels of ‘Costa Rica’ and F3 mature shrunken ‘Tim’ lines (Figure 2a) ranged from 13.9 to 21.9 (Figure 2b). The H* value of ‘Costa Rica’ was significantly lower (*p* < 0.05) than the F3 mature shrunken kernels, except for ‘Tim2’, which was similar (Figure 2b). The lower H* value of ‘Tim2’ was associated with a more purple colouration in comparison to the other purple shrunken genotypes. Significant differences (*p* < 0.05) in H* values were also observed between the ‘Tim’ lines, indicating that colour variation existed between them.

### 2.5. Kernel Anatomy Regarding Anthocyanin Development

**Figure 3:**
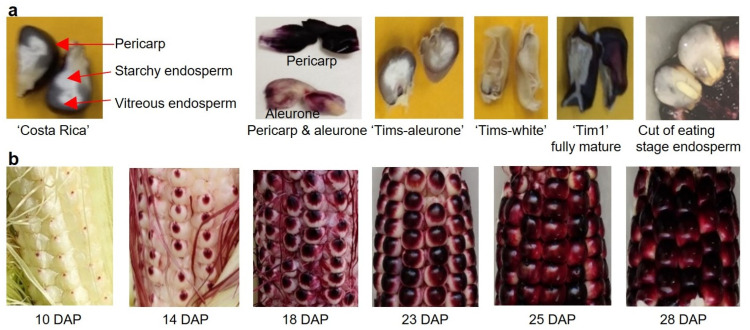
Anatomy of kernels and progression of anthocyanin development. (**a**) Longitudinal sections of kernels (from left): mature ‘Costa Rica’ (showing pericarp, starchy endosperm and vitreous endosperm); separated pericarp and aleurone tissues of the F6 eating stage line ‘Tim1’; sections of fully mature kernels of ‘Tims-aleurone’, ‘Tims-white’ and F3 line ‘Tim1’; cut of a purple sweetcorn kernel at eating stage showing lack of anthocyanin in endosperm (both starchy and vitreous); (**b**) Physiology of anthocyanin development at different stages of kernel maturity (10–28 DAP).

Anthocyanin development in different tissues of the different lines is shown in Figure 3a. The mature ‘Costa Rica’ parental line showed visually distinct anthocyanin pigmentation (purple colour) in the pericarp, aleurone and vitreous endosperm, but not in the starchy endosperm. In contrast, the mature ‘Tims-aleurone’ kernel displayed anthocyanin only in the aleurone layer and vitreous endosperm (outside pericarp was non-pigmented, which was confirmed by visual inspection), and the ‘Tims-white’ parent had no anthocyanin visible in any tissue.

The F3 mature purple shrunken kernels had anthocyanin present in the same tissues as the ‘Costa Rica’ parent. An image of anthocyanin distribution in a mature shrunken kernel of the F3 line, ‘Tim1’ is shown in Figure 3a. The cut mature kernels also showed that there was no visual evidence of anthocyanin development in either the embryo or the starchy endosperm in any of the genotypes assessed. At sweetcorn eating stage (25 DAP), cut kernels of purple sweetcorn indicated that there is no anthocyanin development present in the vitreous endosperm (Figure 3a, last image), in contrast to the mature shrunken kernel.

### 2.6. Kernel Maturity and Anthocyanin Development

A visual inspection of ‘Tim1’ was performed to observe anthocyanin development in kernels at different stages of kernel maturity (Figure 3b). Anthocyanin accumulation was observed to start at the kernel tip (point of attachment of the silk) 10 days after pollination. Anthocyanin accumulation gradually increased from 10 to 18 DAP and started to spread across the surface of the kernel. At 23 DAP, more than three-quarters of the kernel was purple. By 25 DAP, the kernels at the lower part of the cob were fully purple, and some kernels of the upper cob were still turning purple. At 28 DAP, all kernels had turned fully purple. The 25 DAP kernels were used for anthocyanin quantification as representative of sweetcorn eating stage. The kernel moisture content of ‘Costa Rica’, ‘Tim’s white’, ‘Tim1’, ‘Tim2’, ‘Tim4’ and ‘Tim5’ at 25 DAP was calculated to be 68.9%, 70.9%, 72.2%, 72.7%, 74.0% and 70.2%, respectively.

## 3. Discussion

The current research investigated how the anthocyanin profile of novel *shrunken2* purple-pericarp sweetcorn lines varied in comparison to their purple-pericarp ‘Costa Rica’ maize parent. While both ‘Tim1’ and ‘Costa Rica’ had similar total anthocyanin concentration (on a fresh weight basis) at sweetcorn eating stage (Figure 1b), ‘Tim1’ had a greater concentration than ‘Costa Rica’ when fully mature (Table 1). This difference at full maturity can largely be attributed to the lack of production of starch in ‘Tim1’, which leads to the shrunken phenotype as moisture is removed during the maturation process. Consequently, starch has an expected ‘dilution effect’ on total kernel anthocyanin concentration when comparing mature starch with supersweet accessions. Interestingly, the heterozygous (F3) ‘Tim’ lines (‘Tim2’, ‘Tim4’, ‘Tim5’) did have a slightly lower total anthocyanin concentration at 25 DAP than either ‘Costa Rica’ or ‘Tim1’ (Figure 1b). It should not be ruled out that this difference might possibly be linked to a difference in pericarp thickness, as thicker pericarps are commonly selected against in sweetcorn to improve eating texture [21], and these lines may have inherited this trait from the ‘Tims-white’ sweetcorn parent. A reduced pericarp thickness would most likely lead to a lower anthocyanin concentration, despite the presence of aleurone pigmentation, which contributes considerably less to total anthocyanin concentration. In the current trial, it was observed that aleurone-pigmented kernels produced seven-fold less anthocyanin than the pericarp-pigmented ‘Costa Rica’ genotype (Table 1).

The anthocyanin profile of mature kernels was also found to vary between the ‘Costa Rica’ parent and the different ‘Tim’ lines. At full maturity, ‘Costa Rica’ was observed to have the highest ratio (97:3) of purple anthocyanins (cyanidin- and peonidin-based) to red anthocyanins (pelargonidin-based), compared to ‘Tim2’ and ‘Tim5’ (91:9), and ‘Tim1’ and ‘Tim4’ (82:18). Cyanidin-based and peonidin-based together form a purple colour, with previous study reporting that peonidin is formed by methylation of cyanidin [22]. The change in ratio also partly reflects the differences observed in the hue angles of these lines, such that ‘Costa’ is slightly more purple than ‘Tim5’, and that ‘Tim1’ and ‘Tim4’ are a more purplish red colour (Figure 2b). These differences in profile may be partly explained by differences in the alleles of the *red aleurone1* (*pr1*) gene. *Pr1* encodes a flavonoid 3’-hydroxylase enzyme required for the biosynthesis of purple and red anthocyanin pigments [23]. It is possible that the ‘Costa Rica’ parent possesses a homozygous *Pr1Pr1* allele arrangement, resulting in a 97:3 purple/red ratio, while ‘Tim1’ may possess a *pr1pr1* allele arrangement, resulting in an 82:18 ratio. It is interesting that the purple/red ratios did not extend beyond this range, although maize accessions have been identified with a considerably greater percentage of red pelargonidin, such as observed in ‘Apache Red’ [24], indicating there are likely to be multiple alleles of the *Pr1* gene existing in maize. At sweetcorn eating stage (25 DAP), it was observed that the above ratios were relatively maintained. Again, the kernels of the ‘Costa Rica’ parent, and ‘Tim2’ and ‘Tim5’ accessions were observed to have the highest percentage (>90%) of purple anthocyanins, in comparison to ‘Tim4’ (84%) and ‘Tim1’ (78%). This lack of change in the percentage of purple anthocyanins, tends to indicate that increasing kernel maturity has only minimal, if any, impact of the proportion of purple and red anthocyanins present.

The degree of malonylation of anthocyanins was observed to vary both between the ‘Costa Rica’ purple maize parent and within the purple sweetcorn ‘Tim’ lines. Malonylation has been reported to increase stability of anthocyanins which would be of benefit to purple sweetcorn, as sweetcorn is usually cooked prior to consumption. In the present trial, ‘Costa Rica’ and ‘Tim5’ were observed to have the lowest level of malonylation of the lines studied (29–36%), although a survey of purple maize [7] has indicated a high level of malonylation being present in many purple maize varieties. In the present study, however, apart from in ‘Tim5’, malonylation was higher in the ‘Tim’ progeny than in the survey, indicating that this trait may have been inherited from the white sweetcorn parent. Interestingly, the ‘Tim4’ line (87.5%) exceeded the acylated anthocyanin range (2–72%) generated by Chatham and Juvic 2021 [24]. In maize, anthocyanin malonylation has been reported to be controlled by the gene, *anthocyanin acyltransferase1* (*AAT1*) [7]. In the present study, malonylation appeared to be independent of the proportion of cyanidin to pelargonidin, which is largely controlled by *Pr1.* Malonylation has also been reported to be associated with higher total anthocyanin accumulation [24], although this was not apparent in the present study at either 25 DAP or at full maturity (60 DAP).

It was further observed that anthocyanin synthesis was also affected by kernel maturity at harvest, with colour development increasing in conjunction with a progression of anthocyanin development across the kernel surface (Figure 3b). This has been previously reported for *brittle1* sweetcorn [8] and purple maize [25]. Importantly, anthocyanin development occurred during the immature kernel sweetcorn eating stage, such that at 25 DAP, close to the entire kernel surface was purple, giving the cob a more uniformly-coloured appearance, that may assist marketing. Interestingly, although pigmentation was visually apparent in the pericarp and vitreous endosperm tissues of the mature kernels of ‘Costa Rica’ parent and ‘Tim1’, there was no visual sign of pigmentation in the vitreous endosperm at 25 DAP (Figure 3a). In this regard, the development of anthocyanin in the maternal pericarp tissue appears to develop at an earlier stage of maturity than in non-maternal vitreous endosperm tissue. The observation of anthocyanin pigmentation in the vitreous endosperm of mature kernels of purple-pericarp maize also appears to have not been previously reported in the scientific literature, as far as we are aware. It is possible that many purple maize accessions studied previously [7] have had little vitreous endosperm present, and the mainly starchy endosperm present does not appear to accumulate anthocyanin. It is also worth commenting that although pericarp colouration could potentially be increased through harvesting later than 25 DAP, eating quality of the kernel would decline after this point, due largely to moisture loss causing shriveling and a chewier texture [26]. In the present study, the moisture content of ‘Tim’ lines at 25 DAP was within the range of 70–74%, which has been reported previously [27] as being within the optimum moisture content (70–75%) for sweetcorn harvest. This confirms that the 25 DAP kernels in the present study were at sweetcorn eating stage.

Significantly less anthocyanin was produced in ‘Tims-aleurone’ (27.65 mg/100g FW) kernels than in the ‘Costa Rica’ (194.47 mg/100g FW) purple-pericarp kernels at the fully mature stage (Table 1). The reason behind this is that the maternal pericarp tissue consists of several cell layers, while the non-maternal aleurone beneath the pericarp (Figure 3a) consists of single layer of cells [28]. Earlier research by Luna-Vital et al. 2017 [29] has also reported a significantly higher amount of anthocyanin in pericarp-pigmented corn than in aleurone-pigmented corn. The present study would confirm that pericarp-pigmented sweetcorn is a better dietary source of anthocyanin than aleurone-pigmented sweetcorn.

Anthocyanin has different health benefits including the prevention of hypertension as well as different types of cancers [13]. In the current trial, the developed F6 line ‘Tim1’ at eating stage (25 DAP) produced 253 mg/100g FW total anthocyanin. Total anthocyanin concentrations found in red currants, red plums and strawberries have been reported as 12.8 mg/100g FW, 30.1 mg/100g FW and 60 mg/100g FW, respectively [30,31,32]. The current study, therefore, would indicate that purple-pericarp sweetcorn is an excellent source of dietary anthocyanin.

## 4. Materials and Methods

### 4.1. Plant Materials

**Figure 4:**
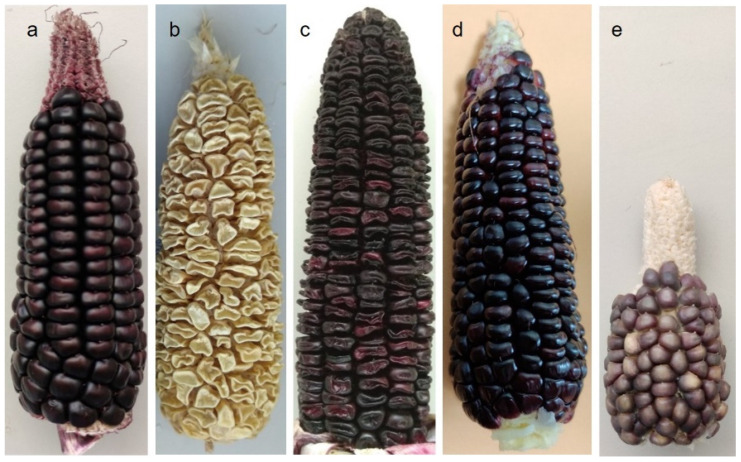
Cobs of parental lines, F3 and F6 progenies, and aleurone-pigmented corn: (**a**) mature purple-pericarp maize, ‘Costa Rica’ (*A1A1.Sh2Sh2*), (**b**) mature white sweetcorn, ‘Tims-white’ (*a1a1.sh2.sh2*), (**c**) mature F3 heterozygous purple-pericarp sweetcorn (*A1a1.sh2sh2*), (**d**) F6 homozygous purple-pericarp sweetcorn (*A1A1.sh2sh2*) at eating stage, and (**e**) mature aleurone-pigmented (non-pericarp pigmented) maize (‘Tims-aleurone’).

Cobs from the purple-pericarp Peruvian maize parental line, ‘Costa Rica’, the white sweetcorn parental line, ‘Tims-white’, and their F3 and F6 progeny (Figure 4) were investigated in the current study. ‘Costa Rica’ is a purple-pericarp starchy maize (*A1A1.Sh2Sh2)* and ‘Tims-white’ is a *shrunken2* (*sh2*) white sweetcorn (*a1a1.sh2sh2*). The F3 progeny developed from these parents was an initial heterozygous purple-pericarp *sh2* sweetcorn line selection (*A1a1.sh2sh2*), while the F6 line ‘Tim1’ was a homozygous purple-pericarp *sh2* sweetcorn line selection (*A1A1.sh2sh2*), and the remainder of the F6 purple sweetcorn lines (‘Tim2’, ‘Tim4’ and ‘Tim5’) were heterozygous (*A1a1.sh2sh2)* for the *A1* gene. Purple aleurone kernels (‘Tims-aleurone’), not related to the purple-pericarp parent or progeny, were also used for comparison of total anthocyanin concentration with the pericarp-pigmented kernels.

Mature kernels at 60 days after pollination (DAP) and at sweetcorn eating stage (25 DAP) were used for the study. Kernels of the parental lines (‘Costa Rica’ and ‘Tims-white’), aleurone-pigmented maize (‘Tims-aleurone’) and F3 progeny at the fully mature stage (60 DAP), were assessed for anthocyanin profile and concentration, while the parental lines were compared with the F6 purple-pericarp sweetcorn lines at sweetcorn eating stage (25 DAP).

### 4.2. Anthocyanin Extraction, Identification and Quantification

#### 4.2.1. Chemicals

Standards for cyanidin-3-glucoside (Cy3G), pelargonidin-3-glucoside (Pg3G) and peonidin-3-glucoside (Pn3G) were purchased from Extrasynthese (Genay, France). Solvents and other chemicals (HPLC or analytical grade) were purchased from Sigma-Aldrich (Sydney, NSW, Australia). Deionized pure water (Millipore Australia Pty Ltd., Kilsyth, VIC, Australia) was utilized in this research.

#### 4.2.2. Anthocyanin Extraction

Approximately 100 kernels were randomly separated from different locations on the cob and immediately snap- frozen using liquid nitrogen. Subsequently, the samples were stored at −20 °C up to three months prior to analysis. Approximately 15 kernels were put into milling vessels and placed in liquid nitrogen for 3 min. The vessels were transferred into a MM400 Retsch Mixer Mill (Haan, Germany) which was operated at 30 Hz for 60 sec. Frozen powdered kernel (subsample, 0.5 g) was accurately weighed and used for the estimation of individual anthocyanin concentration. Anthocyanin extraction from the frozen powder sample was performed according to the methodology of Hong et al. 2020 [8]. Briefly, frozen powdered sample (about 0.5 g) was placed into a falcon tube (50 mL) and 3 mL cold (4 °C) extraction solution (aqueous 80% methanol, 0.1 M HCl) added. A reciprocating shaker (horizontal) RP 1812 (Victor Harbor, SA, Australia) was used to mix the solution at 250 rpm for 10 min at 4 °C under dim light conditions, and then sonicated at 4 °C for 10 min. Centrifugation at 4000 rpm for 10 min (Eppendorf Centrifuge 5804, Hamburg, Germany) was performed at 4 °C. The supernatant was collected, and the pellet reextracted twice following the same process. Combined supernatants were filtered through a 0.20 μm hydrophilic PTFE syringe filter into HPLC vials for subsequent chemical analysis. The extraction was performed in triplicate.

#### 4.2.3. Anthocyanin Identification

Anthocyanin profiles were obtained using UHPLC-DAD-MS (ultra-high performance liquid chromatography–diode array detection-mass spectrometry) (Shimadzu, Kyoto, Japan) as described previously by Hong et al. 2021 [9]. For operation of the instrument and data-processing, LCMS software (Lab solutions) (Ver.5.85; Shimadzu) was used. Chromatographic separation was performed on a reverse phase Acquity UPLC BEH C18 column (100 × 2.1 mm i.d., 1.7 particle size; Waters, Dublin, Ireland). Temperature of the column was maintained at 50 °C and scanning of the DAD spectrum was performed from 200 to 600 nm. The elution was performed with 92% water, 7% acetonitrile and 1% formic acid (100% of mobile phase A) as an initial isocratic hold for 1 min, followed by a linear gradient from 100% to 85% of mobile phase A for 30 min, purging for 3 min at 100% of mobile phase B (acetonitrile, 1% formic acid), conditioning for 1 min, and re-equilibration for 5 min.

#### 4.2.4. Anthocyanin Quantification

Anthocyanins were quantified using a UHPLC–DAD Agilent 1290 Infinity system (Agilent Technologies, Santa Clara, CA, USA; System 2). The UHPLC consisted of a diode-array (DAD) detector (1290 Infinity), pump (1290 Infinity), column oven (1290 Infinity), auto-sampler (1290 Infinity) and a system controller (1290 Infinity). Agilent HassHunter workstation Data Acquisition version B.07.00/Build 7.0.7022.0 was controlled by the UHPLC-DAD system. Agilent MassHunter Quantitative Analysis Version B.07.00/Build 7.0.457.0 was employed for data analysis. Chromatographic separation was achieved on a reversed-phase Acquity UPLC BEH C18 column (150 × 2.1 mm i.d., 1.7 µm particle size; Waters, Dublin, Ireland) at 50 °C with a flow rate of 0.25 mL/min. The DAD spectra were scanned from 200 to 800 nm and anthocyanins were detected at 520 nm. The concentration of individual anthocyanins was determined by calibration curves (external) of Cy3G, Pn3G and Pg3G, and their respective malonated counterparts. To define purple colour, cyanidin and peonidin were pooled together, as peonidin is a methylated form of cyanidin [22].

### 4.3. Objective Colour Measurement

Mature seeds from the round (non-shrunken) ‘Costa Rica’ and F3 purple shrunken progeny (‘Tim1’, ‘Tim2’, ‘Tim4’ and ‘Tim5’) were utilised in this study to identify the impact of total anthocyanin on kernel pericarp colour. Colour was measured objectively [33] for H* (hue angle) with a Chromameter, Konica Minolta CR-400 (Osaka, Japan). Ten kernels were removed from the cob and positioned upright on a black plasticine base to replicate their original placement on the cob.

### 4.4. Statistical Analysis

A 2-sample *t*-test was used to compare the anthocyanins of ‘Costa Rica’ and ‘Tims-aleurone’ at the full mature stage. Prism (GraphPad Prism 9.3.1) statistical software was used for one-way factorial analysis of variance (ANOVA) to assess variances and pairwise multiple comparisons. To compare differences between means, Fisher’s least significant difference (LSD, *p* < 0.05) was used. Three replicates (15 kernels for each sample) were used per cob for statistical analysis.

### 4.5. Kernel Anatomy

To observe the degree of anthocyanin development in the pericarp (outer layers), aleurone layer, or endosperm, a longitudinal section (LS) of mature kernels (60 DAP) of the parents and progeny were visually assessed.

### 4.6. Kernel Physiology

To observe the impact of the stage of kernel development on anthocyanin development, developing ‘Tim1’kernels were visually inspected from 7 to 28 DAP in the field. Photographs were taken to visualize anthocyanin development across the kernel surface.

### 4.7. Moisture Content

Frozen powdered kernels of approximately 1 g was placed in an aluminum dish, weighed, and then placed in a vacuum drying oven (Series RVT, Heraeus, Germany) for 24 h at 70 °C and 33 kPa. The aluminum dish with the dry sample was then allowed to cool down in a glass vacuum desiccator dryer containing silica gel, and the dish containing the dry sample weighed again. An analytical balance (Sartorious CP 234S, d = 0.1 mg, Goettingen, Germany) was used for weighing. Moisture content was determined using the AOAC method 934.01 (AOAC, 1990) and by taking three samples of each sweetcorn line. The following formula was used to calculate moisture content:

(1) $$\mathrm{Moisture}\;\mathrm{content}\;\left(\%\right)=\frac{M_{i}-{\;M}_{f}}{M_{i}}\times 100$$

Here, M_i_ means initial weight and M_f_ means final weight of the powdered sweetcorn kernels.

## 5. Conclusions

The current investigation revealed variation in total anthocyanin content, anthocyanin ratios, and the degree of anthocyanin malonylation between the purple-pericarp maize parent ‘Costa Rica’ and the derived ‘Tim’ *sh2*-supersweet accessions. As with pericarp pigmented maize, increase in anthocyanin in the ‘Tim’ accessions was associated with a progressive spread of pigmentation across the kernel surface. At sweetcorn-eating stage (25 DAP), most of the visible kernel surface was pigmented, with anthocyanin concentration reaching as high as 253 mg/100g FW. Although anthocyanin concentration was observed to further increase with increasing kernel maturity, more mature sweetcorn kernels have reduced eating quality primarily due to a reduction in textural quality.

Kernel colour (hue angle) appeared to be closely associated with the ratio of purple anthocyanins (cyanidin- and peonidin-based anthocyanins) to red anthocyanin (pelargonidin) present in kernels, although cyanidin-based anthocyanins always accounted for the majority of anthocyanin present, and as such, kernel colour only varied from a reddish-purple to purple colour. It was interesting to note that anthocyanin pigmentation was not only present in the pericarp and aleurone tissue at sweetcorn-eating stage, but also in the vitreous endosperm of fully mature kernels.

The variation in anthocyanin parameters observed in the derived sweetcorn accessions is likely to have been inherited from their white sweetcorn parent. This potentially enables the development of slightly different coloured sweetcorn varieties (purple vs. reddish-purple), but also varieties with potentially greater anthocyanin stability due to a higher proportion of anthocyanin malonylation. This is of particular relevance to sweetcorn, which is usually cooked prior to consumption. The development of purple-pericarp sweetcorn provides a novel high-anthocyanin vegetable to the marketplace, which may provide additional health-benefits in comparison to standard yellow/white sweetcorn.

## Figures and Tables

**Table 1:** Total and individual anthocyanin concentrations at 60 DAP of the mature round (starchy) parental line (‘Costa Rica’), sweet white parental line ‘Tim’s white’, and aleurone pigmented ‘Tim’s-aleurone’ round kernels at 60 DAP; and the ‘Tim’ purple sweetcorn segregants of the F3 purple-shrunken kernels.

	Anthocyanin-Based Compound (mg/100g FW)			
Accession (60 DAP)	Cyanidin	Peonidin	Pelargonidin	Total Anthocyanin
‘Costa Rica’ ^A^	160.5x	27.1x	6.7x	194.5x
‘Tims-aleurone’	15.8y	9.0y	2.9x	10.3y
‘Tims-white’	n.d.	n.d.	n.d.	n.d.
‘Tim1’ ^B^	230.1a	68.4b	66.9b	365.4a
‘Tim2’	813.5c	197.5d	98.3c	1109.3c
‘Tim4’	225.7a	50.3a	59.4ab	335.3a
‘Tim5’	506.9b	85.7c	56.8a	649.3b

Means within columns followed by the same letter are not significantly different (*p* < 0.05); ^A^ Mean separation based on 2-sample *t*-test; ^B^ Mean separation based on one-way analysis of variance (ANOVA); n.d. = not detected.

**Table 2:** Cyanidin-, peonidin- and pelargonidin-based anthocyanins and their proportions (based on percentage of total peak area) in the F6 ‘Tim’ purple-pericarp sweetcorn accessions and their parental line, ‘Costa Rica’. The peak number indicates the order of elution as shown in Figure 1a.

		Anthocyanin Proportion (% Total Peak Area)				
Anthocyanin	*m*/*z* (Peak No.)	‘Costa Rica’	‘Tim1’	‘Tim2’	‘Tim4’	‘Tim5’
Cy3G	449.2 (1)	54.8	15.5	19.5	6.9	60.9
Cy3MG	535.2 (4)	20.9	30.7	34.3	27.9	15.2
Cy3DMG	621.2 (6)	3.7	24.7	27.4	28.3	5.1
Pn3G	463.2 (3)	3.3	3.7	6.6	3.0	3.8
Pn3MG	549.2 (7)	5.5	1.7	2.7	2.4	3.4
Pn3DMG	635.1 (9)	2.4	1.4	2.6	15.2	2.4
Pg3G	433.2 (2)	5.9	7.5	1.7	2.6	5.8
Pg3MG	519.1 (5)	2.1	12.7	3.9	8.6	2.1
Pg3DMG	605.2 (8)	1.4	2.1	1.4	5.1	1.2

## Data Availability

All data is presented in the tables and figures. However, raw data could be available upon request to the corresponding author A.A.

## Sample Availability

Samples of the purple maize, purple- and white sweetcorn are available upon request to the corresponding author T.J.O.
